# Biological monitoring of occupational exposure to inorganic lead: a comparison between salivary, blood and airborne lead levels

**DOI:** 10.1186/s12940-025-01246-8

**Published:** 2025-12-24

**Authors:** Loreta Tobia, Riccardo Mastrantonio, Mario Muselli, Lorenzo De Rossi, Elio Tolli, Vincenza Cofini, Elena Grignani, Angela Gambelunghe, Marco Dell’Omo, Stefano Necozione, Leila Fabiani

**Affiliations:** 1 Department of Life, health and environmental science, University of L’Aquila, L’Aquila, Italy; 2 Environmental Research Center, Istituti Clinici Scientifici Maugeri IRCCS, Pavia, Italy; 3 Department of Medical and Surgical Sciences, Alma Mater Studiorum, University of Bologna, Bologna, Italy; 4 Department of Medicine, University of Perugia, Perugia, 06123 Italy

**Keywords:** Lead, Occupational exposure, Biomonitoring, Saliva, Biological matrix, Blood lead, Industrial hygiene

## Abstract

**Background:**

Inorganic lead exposure is a relevant occupational health issue in many industry sectors. European regulations set specific limits for time-weighted chemical airborne exposure and for biological exposure. While blood lead monitoring is the current standard, saliva sampling offers a less invasive alternative for biomonitoring. This study evaluates the potential of salivary lead assessment as an alternative biological matrix for occupational exposure monitoring.

**Methods:**

An observational study was conducted at a lead-acid battery production facility in Central Italy from July to December 2024. Ninety-two male workers participated: 46 occupationally exposed and 46 non-exposed workers. Salivary lead levels were measured using ICP-MS in all participants. Blood lead levels and personal airborne lead assessments were performed in the exposed group. Socio-demographic data were collected through self-administered questionnaires.

**Results:**

Mean salivary lead levels were significantly higher in exposed workers (23.3 ± 41.4 ng/swab) compared to non-exposed workers (0.3 ± 0.6 ng/swab, *p* < 0.001). A moderate positive correlation was found between environmental and salivary lead levels (ρ = 0.5587, *p* = 0.0003). No significant correlations were observed between blood and salivary lead levels or between environmental and blood lead levels. Occupational exposure and alcohol consumption were significant predictors of salivary lead levels.

**Conclusions:**

Saliva appears to be a promising alternative matrix for recent lead exposure monitoring, showing better correlation with environmental exposure than blood lead levels. Further research is needed to establish reference values and standardize salivary lead biomonitoring protocols.

**Supplementary Information:**

The online version contains supplementary material available at 10.1186/s12940-025-01246-8.

## Background

Inorganic lead represents a public and occupational health concern. Its environmental full life cycle consists of migration, accumulation, stability, and organic reaction: it can indeed be easily transferred and enriched through food chains and drinking water, threatening human health [1]. The related adverse health effects consist in organ-specific toxicity and toxicity to the reproductive system. Similarly to general chemicals, human exposure is possible by three main pathways: direct ingestion, inhalation and dermal contact [2]. For instance, lead exposure is associated with adipogenic and endocrine disrupting, mostly if combined with other inorganic pollutants [3]. In addition, it can play a role in pathogenesis and severity of rheumatic and non-communicable diseases [4, 5].

The European Community recently set lower environmental and biological limit values. After a transitional period that will last until 2028, the new occupational exposure limit is set as 0,03 mg/m^3^ (time weighted average, 8 h). Regarding the biological exposure index (BEI) for blood, the new limit value is set as 30 µg Pb/100 ml. The European Directive requires the employer to organize health surveillance if the detected environmental and biological exposure levels exceed 0.015 mg/m^3^ or 9 µg Pb/100 ml [6]. Moreover, for female workers in childbearing age, the lawmaker requires that medical surveillance is carried out when reference lead values of the general population are exceeded. If such values do not exist, medical surveillance is carried out when hematic lead values exceed 4,5 µg/100 ml.

Human lead intake may be influenced by hand-to-mouth activities and by inadequate oral hygiene behavior [7, 8]. Occupational exposure to inorganic lead or its compounds may lead to bioaccumulation in target organs and to chronic effects. Many studies highlight that occupational tasks performed by artisans [9] and industrial workers [10, 11] are undoubtedly associated with high blood lead levels.

An exogenous chemical substance or its metabolite in an organism may be identified through the analysis of biological matrices. In epidemiological investigations, biomarkers are frequently employed to assess heavy metals exposure and calculate internal dose. A recent review [12] points out that lead exposure can be assessed through blood, bone, teeth, nails, hair and urine analyses: among them, good biomarkers are represented by whole blood, bone and teeth. Salivary biological matrix can be considered a blood surrogate, particularly with regard to the plasma compartment [13]. Saliva sampling could be preferred to blood sampling due to its lower invasiveness.

The main aim of the present study is to evaluate the potential of salivary lead assessment as an alternative biological matrix for occupational exposure to lead. We also aim to monitor occupational lead exposure through salivary lead assessment and to evaluate possible associations with individual socio-demographic variables. Moreover, in a sub-sample we compared the salivary results with blood lead and airborne values. We sought to compare the salivary lead values and the blood lead values and relate them to the actual environmental exposure.

## Materials and methods

This observational study was conducted in 2024, from July to December, at a lead-acid battery production facility in Central Italy. It leverages data collected from the plant’s mandatory occupational health surveillance. While exposed employees undergo periodic blood lead monitoring and airborne lead monitoring as required by the health surveillance program and the occupational risk assessment, we supplemented this routine surveillance with two voluntary study components:

1. A comprehensive self-administered questionnaire assessing:
  Socio-demographic characteristics.
  Lifestyle factors (tobacco/alcohol use, physical activity).
  Onychophagia.
  Occupational history and work practices.
2. Saliva collection using sterile synthetic swabs.

**Fig. 1:**
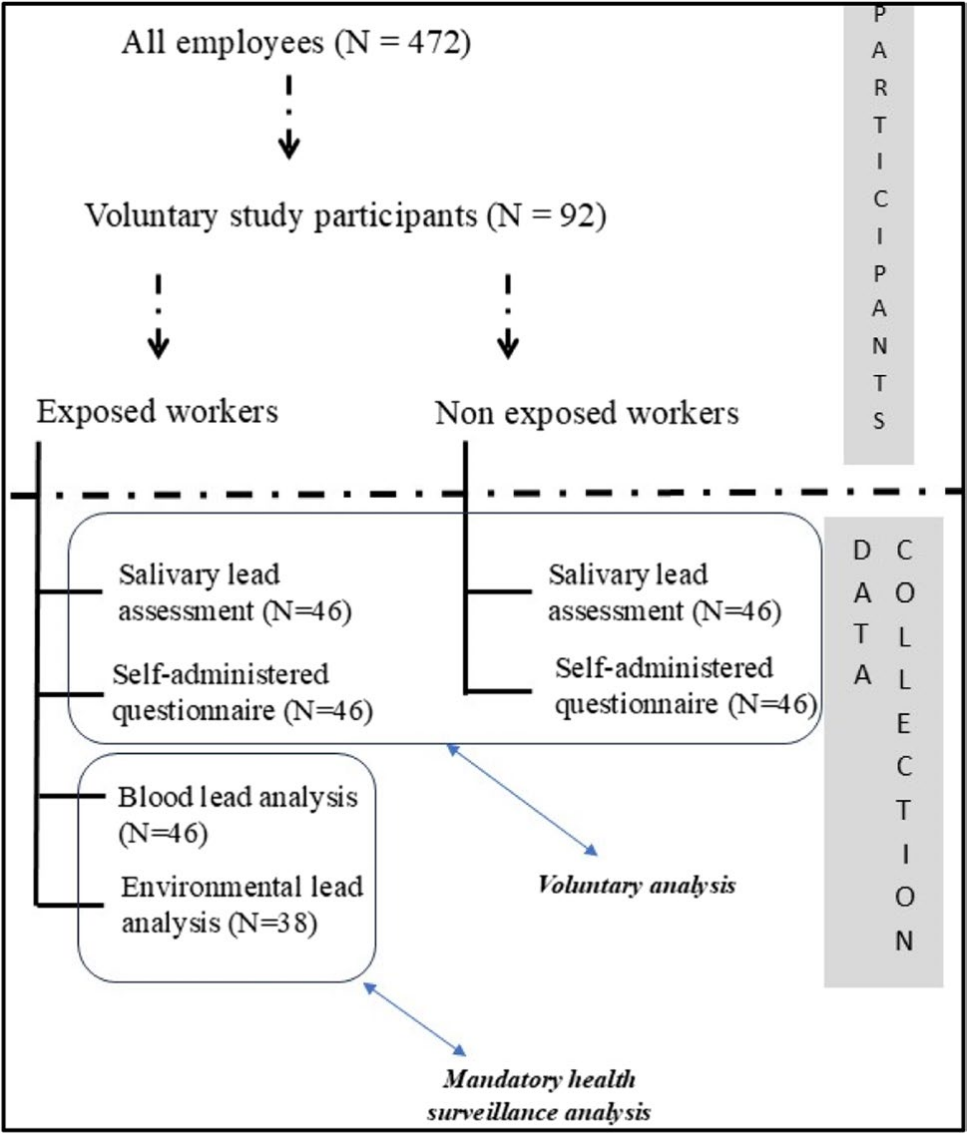
- Inclusion criteria and data collection

Inclusion criteria and the performed analysis for each group are displayed in Fig. 1.

Out of a total of 472 workers, 96 chose to undergo salivary sampling and complete the questionnaire (response rate 20.34%). Based on self-reported job tasks verified with plant records, participants were classified as:

- Exposed group (*n* = 46): Workers with direct exposure to inorganic lead who work in an environment where lead is recognized as an airborne pollutant (e.g. those working in the production department of the company).
- Non-exposed group (*n* = 46): Administrative staff with no occupational lead exposure.

The 46 exposed workers underwent blood lead analysis and airborne personal lead assessment as mandated by law for workers with potential lead exposure. Blood lead levels were obtained from the existing surveillance system data. Thirty-eight exposed workers underwent personal airborne lead assessment. The data presented in this study partially stem from a voluntary participation and partially from mandatory health surveillance assessment (Fig. 1). Among all the workers that underwent environmental lead monitoring, we were able to select only 38, as the others did not consent to participate in the voluntary part of the study. The exposed workers belong to the same Similar Exposure Group (SEG) as they all belong to the job activity “production operator”, with no differences in areas or in the activities performed inside the plant. For the totality of the exposed participants, respiratory personal protective equipment (FFP2 or FFP3) is mandatory.

Both the exposed and the non-exposed groups underwent salivary lead and socio-demographic characteristics assessment. A detailed description of the performed analysis is reported below.

No matching or pairwise selection was performed between groups.

Saliva and blood sampling and lead quantification were performed in July 2024.

### Quantification of the environmental airborne lead concentrations

The investigation was carried out in accordance with the relevant technical standards [14–16], and in cooperation with the Company Managers. The sampling involved the company’s production department, and it was carried out under the regular working conditions. SEGs were identified in accordance with the UNI EN 689:2019 standard [16]. Active samplers were used to measure concentrations in the worker’s breathing zone and the results were compared with the relevant Occupational Exposure Limit Values (OELVs).

Sampling of the inhalable dust fraction was performed on a cellulose ester membrane filter, diameter 25 mm, 0.8 μm pore size, by using the IOM (Institute of Occupational Medicine) sampler. The quantification of the inhalable dust fraction was performed by using a weighting technique after conditioning the filter membrane, before and after sampling, at constant temperature and humidity [17]. Lead concentrations in the sampled inhalable dust fraction were measured by using acid digestion and Inductively Coupled Plasma-Mass Spectrometry (ICP-MS) (in-house method derived from NIOSH 7303) [18] using the Perkin Elmer Nexion 2000.

### Quantification of saliva lead levels

Saliva lead levels were measured by using standard salivary swabs and cryogenic vials stored for few hours at a temperature of 4 °C and then frozen at −20 °C to ensure secure and contamination-free storage of the sample. The technique performed by the specialist involved the introduction of the swab into the oral cavity using small circular motions (for at least 30 s) around the floor of the mouth and into Wharton’s duct and the lingual caruncles. Salivary lead was measured by using ICP-MS at ng/swab (Quadrupole with collision cell. Mod. NEXION 2000 B). Quantification is performed by preparing standard solutions in 1% nitric acid with known lead concentrations, with an analytical limit set at 0,2 ng/swab (limit of detection) (analytical detection masses: Pb206, Pb207, and Pb208). Internal standardization with holmium (Ho) corrects for any instrumental drift and matrix effects. Internal quality controls were performed for the following concentrations: 10 ng/L Pb (recovery percentage 80–120); 10 µg/L Pb (recovery percentage 80–120).

### Quantification of blood lead concentrations

Blood lead concentrations were measured by using ICP-MS, an elemental analysis technique that offers high sensitivity and very low limit of detection (LOD) at or below a part per trillion (10 − 12 ppt), based on a 98% confidence level. Blood samples were collected in EDTA heparinized tubes. Analyses were carried out in accordance with the ISTISAN 15/30 method [19]. Mean blood lead values are reported in the tables expressed in µg/dL ± standard deviation (SD).

### Assessment of the socio-demographic characteristics of the participants

After obtaining informed consent, and in compliance with the privacy and sensitive data protection rules, a questionnaire was self-administered both to the exposed and the non-exposed workers. Based on relevant Literature, the aim of the questionnaire was to collect information about age, smoking and alcohol consumption [11], physical activity [13], Body Mass Index (BMI), medications and seniority [20]. Based on the findings of Wu et al. [20], PPE use, training attendance and onychophagia were assessed among the exposed workers.

### Statistical analysis

Quantitative variables are given with mean and standard deviation, qualitative variables are given with absolute and relative frequencies. Qualitative variables were compared by using the chi-square test or the Fisher’s exact test, as appropriate. After conducting the Shapiro-Wilk test for normality, the quantitative variables were compared using the Mann-Whitney and Kruskal-Wallis tests. Spearman’s correlation was used to assess the association between quantitative variables. Finally, a multivariate linear regression model was designed to investigate possible predictors of the salivary lead levels. For each analysis, an alpha level of 0.05 was considered to be statistically significant. The statistical analysis was performed with STATA 17 software for Windows.

## Results

The study involved 92 male subjects employed in the same enterprise, of which 46 occupationally exposed to lead. Table 1 shows the distribution of the variables analysed through the questionnaire administered to both the exposed and the non-exposed workers.

**Table 1:** – Characteristics of the sample

Characteristics	*N* or mean (% or SD)
Occupationally exposed	
No	46 (50.0%)
Yes	46 (50.0%)
Age (years, range)	
< 30	15 (16.5%)
30–39	17 (18.7%)
40–49	21 (23.1%)
50–59	23 (25.3%)
> 60	15 (16.5%)
BMI (Kg/m ^2^)	25.9 ± 3.2
Alcohol consumption	
No	44 (47.8%)
Yes	48 (52.2%)
Sport	
No	52 (58.4%)
Yes	37 (41.6%)
Onychophagia	
No	76 (84.4%)
Yes	14 (15.6%)
Medications	
No	60 (67.4%)
Yes	29 (32.6%)
Smoker	
No	49 (53.3%)
Yes	40 (46.7%)

Table 2 Shows the results of the association between salivary lead values and each variable analysed in the questionnaire with respect to the whole sample (exposed and not-exposed groups). A statistically significant association was found with the variables “alcohol consumption”, and “occupational exposure”. The average value of lead in saliva of the non-exposed group was significantly lower (0.3 ± 0.6 ng/swab) than in the lead-exposed workers (23.3 ± 41.4 ng/swab). Spearman’s rank correlation revealed no association between BMI and salivary lead (rho= −0.0138; *p* = 0.8962).

**Table 2:** Association of salivary lead levels with the characteristics of the whole sample (exposed and non-exposed workers N=92)

Characteristics (*N* = 92)	Salivary Lead ng/swab (Mean ± SD)	*p*-value
Age (years, range)		0.409
< 30 (15)	12.3 ± 27.4	
30–39 (17)	25.6 ± 57.9	
40–49 (21)	6.5 ± 13.7	
50–59 (23)	12.6 ± 26.3	
> 60 (15)	2.6 ± 7.7	
Smoker		0.729
No (49)	11.2 ± 23.7	
Yes (40)	13.4 ± 39.8	
Alcohol consumption		**0.005**
No (44)	7.26 ± 19.34	
Yes (48)	15.97 ± 38.95	
Sport		0.677
No (52)	13.1 ± 36.5	
Yes (37)	7.5 ± 1.7	
Onychophagia		0.319
No (76)	14.1 ± 34.0	
Yes (14)	1.1 ± 1.4	
Medications		0.668
No (60)	13.2 ± 3.8	
Yes (29)	7.2 ± 15.6	
Occupational exposure		**< 0.001**
No (46)	0,3 ± 0.6	
Yes (46)	23.3 ± 41.4	

Table3 reports the results from the multivariate regression model in relation to the possible predictors of salivary lead levels. Occupational exposure to inorganic lead (p = 0.001) seems to be the only possible predictor of salivary lead levels. The model shows a goodness of fit (R^2^) of 0,150 and a significance level of 0,003.

**Table 3:** Linear regression of possible predictors of salivary lead levels

Variable	Estimate	95% CI	*p*-value
Exposure			
Non-exposed workers			
Exposed workers	23.55	9.59, 37.51	**0.001**
Alcohol consumption			
No			
Yes	−0.99	−14.70, 12.73	0.886
Onychophagia			
No			
Yes	−5.73	−23.81, 12.36	0.531

A more specific statistical analysis regarding factors influencing salivary lead levels is provided in the supplementary material. We observed that most of the non-exposed group reported a very low frequence of alcohol consumption. Conversely, the exposed group show higher percentages of high frequence in alcohol consumption. Due to this behavioural aspect, a Sperman’s rank correlation showed a positive correlation between salivary lead and alcohol consumption in the total sample (exposed and non-exposed workers). Such correlation appears to be moderate and statistically significant (Rho = 0.372, *p* < 0.001). This may explain the significant association between salivary lead levels and alcohol consumption.

Table4 reports the results of the association between average blood lead levels and average salivary lead levels and the investigated variables in the exposed group, respectively. Mean blood lead levels values are expressed in µg/dL; mean salivary lead levels are expressed in ng/swab.

**Table 4:** Association of mean blood lead levels and mean salivary lead levels in the exposed group (N = 46) with the socio-demographic characteristics

Characteristics (*N*)	Blood lead (µg/dL) Mean ± SD	*p*-value	Salivary lead (ng/swab) Mean ± SD	*p*-value
Age (years, range)		0.846		0.308
< 30 (9)	11.16 ± 4.66		22.89 ± 34.99	
30–39 (9)	10.79 ± 6.46		47.88 ± 74.32	
40–49 (10)	13,16 ± 9.87		13.15 ± 17.87	
50–59 (13)	14.21 ± 6.53		22.18 ± 32.19	
> 60 (6)	17.00 ± 12.60		6.11 ± 11.91	
Smoker		0.786		0.777
No (25)	13.39 ± 6.20		21.66 ± 29.75	
Yes (21)	12.73 ± 9.79		25.19 ± 52.74	
Alcohol consumption		0.653		0.661
No (12)	11.35 ± 5.77		25.84 ± 30.72	
Yes (34)	13.45 ± 8.34		22.36 ± 44.90	
Sport		0.356		0.222
No (23)	14.48 ± 7.46		29.23 ± 50.97	
Yes (20)	12.23 ± 8.22		13.61 ± 25.51	
Onychophagia		0.238		0.365
No (41)	13.04 ± 7.75		25.89 ± 43.14	
Yes (3)	19.85 ± 11.10		2.82 ± 2.05	
Medications		0.732		0.307
No (28)	13.79 ± 6.76		27.78 ± 50.44	
Yes (15)	12.90 ± 9.80		13.71 ± 19.74	
Use of PPE		0.080		0.323
No (3)	5.43 ± 5.80		0.53 ± 0.53	
Yes (42)	13.66 ± 7.74		25.43 ± 42.71	
Seniority		0.402		0.683
< 1 (2)	10.50 ± 4.95		26.66 ± 37.70	
1–5 (13)	10.60 ± 5.57		35.26 ± 65.02	
5–10 (5)	18,06 ± 8,09		36.94 ± 36.65	
> 10 (26)	13.52 ± 8.69		14.39 ± 2.,18	
Training programme		0.604		0.635
No (5)	11.36 ± 9.67		14.85 ± 19.84	
Yes (41)	13.33 ± 7.72		24.30 ± 43.32	

Spearman’s rank correlation revealed no association between BMI and blood lead (rho=−0.0138; *p* = 0.8962) and between BMI and salivary lead (rho=−0.0173; *p* = 0.9085) in the exposed group.

**Fig. 2:**
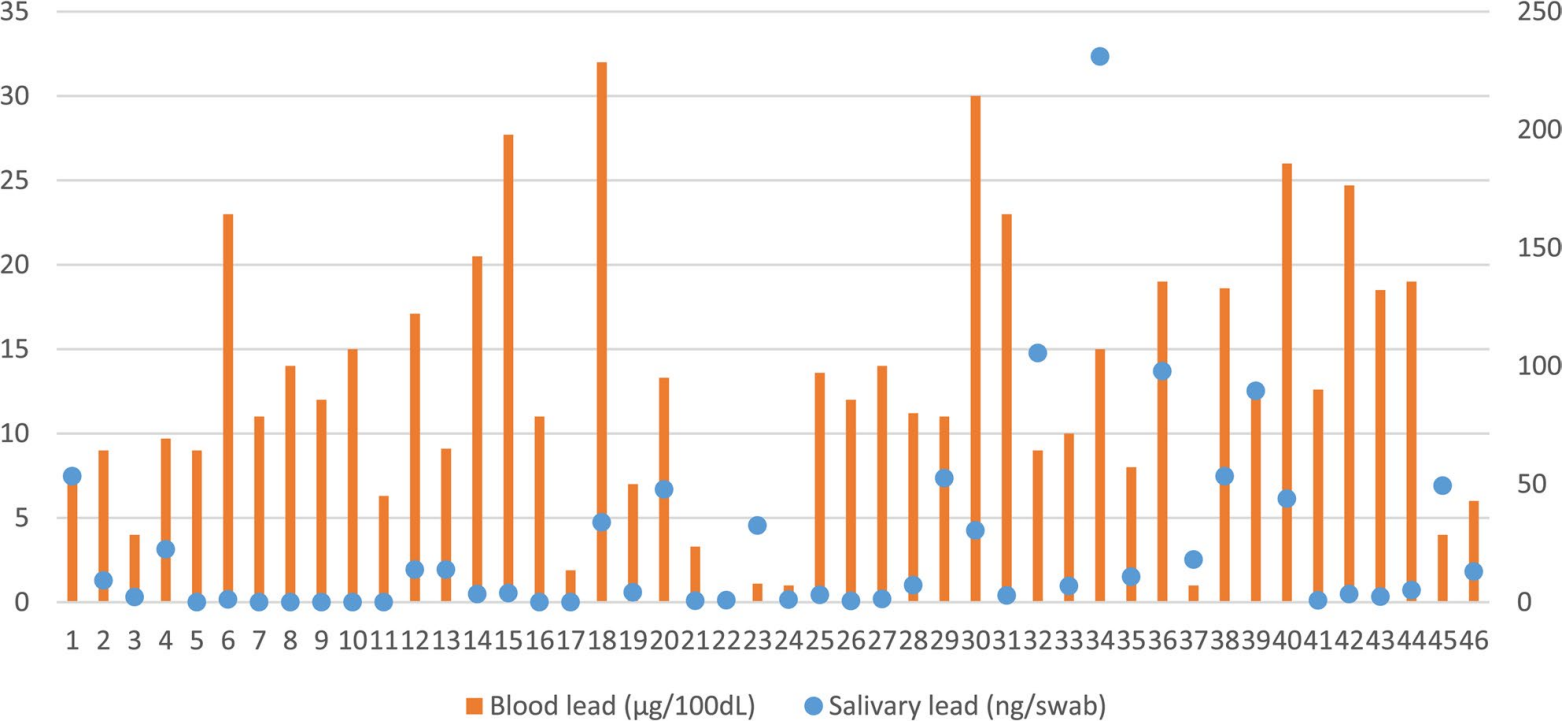
- Blood and salivary lead levels in the exposed workers (*n* = 46). Blood lead levels ranged from 1 to 32 µg/dL (left axis); salivary lead levels ranged from 0,22 to 231 ng/swab (right axis)

Figure 2 illustrates the individual comparison between blood and salivary lead levels across all exposed workers, revealing distinct and independent exposure patterns. The data demonstrate a clear lack of correlation between the two biological matrices, with salivary lead showing substantially greater inter-individual variability (ranging from 0 to 231 ng/swab) compared to blood lead levels (0–32 µg/dL).

**Fig. 3:**
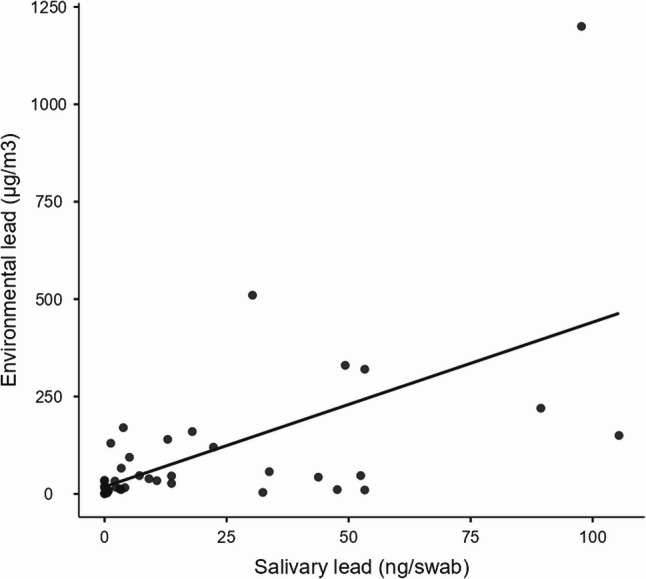
- Association between environmental lead and salivary lead levels. Due to the restricted number of the environmental lead quantification, the reference sample size for this chart amount to 38

Thirty-eight exposed workers underwent personal airborne lead assessment. The average lead concentration in the worker’s breathing zone of the production department was 125.83 ± 172.65 µg/m^3^. The scatter plot in Fig. 3 shows that a positive correlation was found between the environmental lead concentrations and the salivary lead levels for each exposed worker.

Finally, as shown in Table 5, a moderate correlation between environmental lead levels and salivary lead levels was found. The table also reports the absence of a correlation between blood lead and environmental lead and between salivary lead and blood lead levels.

**Table 5:** Correlation between environmental, salivary and blood lead levels

Environmental lead and salivary lead levels	Spearman’s coefficient	0.5587
	p-value	**0.0003**
Environmental lead and blood lead levels	Spearman’s coefficient	0.2663
	p-value	0.1059
Salivary lead and blood lead levels	Spearman’s coefficient	0.1162
	p-value	0.4856

## Discussion

The aim of this study is to identify alternative biological matrices to blood for monitoring occupational exposure to inorganic lead.

Variables such as age, BMI, smoking, use of medications, onychophagia and sport do not appear to significantly affect the differences between salivary lead levels in the involved participants. Conversely alcohol consumption and occupational exposure show a significant association with salivary lead values, especially when we tested the correlation with alcohol consumption (frequence). Limited to these factors, therefore, there is a compliance with the study by Vives et al. [21]., in which a positive correlation was found between biological lead levels and alcohol consumption. Moreover, our findings align with the results of the study by Grandjean et al., [22] conducted on a Danish population of 1052 individuals; they highlighted a strong influence of ethanol on lead levels, even greater than that of cigarette smoke, which is known to contain lead and other metals among many harmful substances. Despite we identified an association between alcohol and salivary lead (Table 2 and supplementary material), it disappeared when tested in the multivariate regression model, probably due to the limited sample size.

As confirmed by our analysis, occupational exposure is known to be a possible predictor of higher salivary lead levels [23].

Our results show that the blood lead levels of each worker sampled largely comply with the law limits set out in Legislative Decree 81/08 [24] as amended by Legislative Decree 135/2024 [25], which came into force last 11 October and implements EU Directive 2022/431 [26] of the European Parliament, according to which the biological limit value must not exceed 60 µg Pb/100 ml in blood. Although the legal limits for blood lead levels in our sample are currently met, the European Community [6] has planned to lower these limits in the near future. Starting April 2026, the limit will be lowered to 30 µg/dl and then reduced by half in early 2029. Environmental exposure levels will also be lowered to 0,03 mg/m^3^. The current limit value is set to 0,15 mg/m^3^. These measures aim to enhance worker protection and will necessitate companies to adopt robust prevention and protection strategies. Limited to the involved exposed workers, average exposure value appear to be lower than the current limit value.

Limited to the exposed group, we analysed the association of blood and salivary lead levels with other variables. Neither blood lead levels nor salivary lead levels appeared to be associated with other variables.

The mean salivary lead levels of the non-exposed group are far lower than those of the exposed group. Such finding may suggest that occupational exposure is a predictor for higher salivary lead levels. Our regression analysis confirms this hypothesis. No further statistically significant associations were found. Our results partially match with the findings from the NHANES study [27], in which the authors highlighted that occupation characteristics may explain exposure to toxics, especially in blue-collar populations. Despite this match, the studied population shows low mean blood lead values compared to ours (µ < 3 µg/dL). Furthermore, in the cited study, the authors also identified a significant contribution of other variables such as age and smoking in biological levels of many pollutants, including lead. Our analysis doesn’t confirm such contributions.

As shown in Table 5, we identified a moderate positive correlation between environmental and salivary lead levels. This finding suggests that saliva may represent a reliable matrix for the recent lead contamination estimation. On the contrary, no significant correlations were found between blood and salivary lead levels, nor between environmental and blood lead levels. It is known that lead accumulate in bone and teeth and that blood levels may change depending on the metal skeletal mobilization [28]. In light of this, the lead body burden evaluation by using the hematic matrix may be influenced by such phenomena. The use of the salivary matrix, instead, may help to assess the current exposure to inorganic lead. The absence of a correlation between salivary and blood lead levels that resulted from our regression analysis is partially confirmed by the findings of P’an [29]. In his study he highlighted a moderate correlation between blood and salivary lead, but he also found that the increasing trend of lead levels in the two matrices in the studied groups was not parallel. Anyway, the cited study involved a bigger sample of exposed and non-exposed workers (a total of 266) in comparison to us and reported salivary and hematic lead levels far exceeding ours, on average.

Our findings align with the views of some authors such as Klotz et al., P’an, Claymaet et al., and Morton J. et al. [13, 29–31] who support the validity of the salivary biological matrix for monitoring inorganic lead exposure. Notwithstanding this concordance, some setting-specific discrepancies must be highlighted. A correlation between salivary and blood lead levels is found only for exposures above the exposure limits [13]. The study of Cleymaet et al. [30] is focused on a very small sample (23) of general population meanwhile Morton et al. [31] reported findings from a working population (85) but an environmental exposure assessment and information of a non-exposed group are not provided. All the cited studies [13, 30, 31] adopted a different salivary collection procedure with respect to us. Given this, our results are partially in line with the opinion of other authors such as Staff et al., Koh D. et al., Barbosa et al., Michalke *et a*l. and Gil et al. [32–36].,. They also found a weak correlation between the lead levels found in the salivary matrix and the levels found in the blood of the same exposed workers. In light of this, they do not support the use of the salivary biological matrix for monitoring inorganic lead exposure. In contrast to our study, however, their studies are missing data on environmental exposure. Moreover, some studies [32, 33, 36] did not involve a non-exposed group. Coherently with our sample, the involved exposed populations were occupational exposed. Supporting data that has already been observed, some literature reviews [34, 35] highlighted that results concerning lead salivary values are affected by significant intra-study variability. This characteristic also affected our results.

In contrast to the studies already discussed [13, 29–36], in which salivary lead analysis involved a volume of saliva and results are expressed as concentration (mass/volume), our salivary sampling consisted in the use of a swab in the oral cavity of the participant. Given that an official method for lead analysis in the salivary matrix is missing, our choice to perform a swab-based sampling is grounded on an easier and mostly faster sampling procedure. This procedure might allow a fast collection even for large numbers of workers, considering that “traditional” saliva collection may require up to ten minutes/worker [31]. To the best of our knowledge, there are no existing studies based on swab-based salivary lead sampling. In this sense, our results can represent a strong point in support of this methodology and a specific study aimed at validating the method would be of great interest.

Salivary lead estimation may be influenced by oral contamination. Despite this, we found that most authors performed salivary sampling without a previous mouth rinsing [30–32, 35]. This procedural characteristic fully aligns with our procedure and, as already discussed, their results also align with ours in terms of salivary lead reliability for biomonitoring [30, 31] and in terms of the absence of a correlation between hematic and salivary lead levels [32, 35]. Conversely, P’An [29] and Koh et al. [33] performed a pre-sampling mouth rinse with water but their findings completely disagree. P’An found that saliva may be considered a good matrix meanwhile Koh et al. concluded that saliva is not a reliable matrix for biological monitoring of lead exposed individuals. Despite our findings globally aligns with several cited studies, oral contamination appear to be an underinvestigated issue, and specific studies are needed to shed light on the topic.

The present study has some limitations. The small sample size could affect the identification of associations or correlations between the matrices analysed as well as between the blood matrix analysed and the environmental exposure. The study’s occupational setting limits the generalizability of the results due to the sample being from a single company, lacking female workers, and having a low response rate due to voluntary participation.

A further limitation is the absence of quantification of the blood lead levels in the non-exposed group. Furthermore, only 38 of the exposed workers underwent personal air sampling; as a result of this, there is a discrepancy between the number of biological samples collected from workers and the number of air measurements performed. The correlation between environmental lead concentrations and salivary lead may be influenced by the contamination of the oral cavity. Our strategy does not allow us to assess the possible contribution of such contamination to the amount of salivary lead and it also lacked in assessing further contamination sources like food or leisure time activities.

Lastly, the implemented sampling method do not allow us to directly compare salivary lead levels with those reported in other studies, expressed as lead concentration in saliva.

## Conclusion

In Italian regulation, the mandatory biomarker for occupational lead exposure is lead concentration in whole blood. Our study points out that saliva may be considered as an alternative matrix for occupational monitoring. In support of this argument, our results show that salivary lead levels appear to be influenced by airborne lead levels.

Based on what stated so far, the effectiveness of the method applied in this study for the quantification of salivary lead levels deserve to be further investigated. Future research should focus on confirming or confuting our findings, for example involving a bigger sample, also considering the conflicting information available in Scientific Literature. Biomonitoring practice needs for standardized protocols and reference ranges with regard to salivary lead assessment.

## Supplementary Information

Supplementary Material 1.

## Data Availability

The datasets used and/or analysed during the current study are available from the corresponding author on reasonable request.

## Abbreviations

BEI: Biological exposure index
BMI: Body mass index
ICP-MS: Inductively coupled plasma mass spectrometry
IOM: Institute of Occupational Medicine
LOD: Limit of detection
OELVs: Occupational Exposure Limit Values
PPE: Personal protective equipment
PPT: Part per trillion
SEG: Similar Exposure Groups
SD: Standard deviation
