# Supplementary material for: Biological monitoring of occupational exposure to inorganic lead: a comparison between salivary, blood and airborne lead levels
Source: Environ Health. 2025 Dec 24;24:95. doi: 10.1186/s12940-025-01246-8 (PMC12729134; doi:10.1186/s12940-025-01246-8)
Supplement: Supplementary file 1 — Supplementary Material 1. [file 12940_2025_1246_MOESM1_ESM.docx]

This supplementary material shows information related to the whole sample (exposed and non-exposed workers (N=92)). This analysis is based on the search for associations between data on frequency of alcohol consumption (never, occasionally, 2-3 times a week and daily), quantitative smoking data (no smoker, <10 cigarettes/day, between 11 and 20 cigarettes/day and more than 20 cigarettes/day) and salivary lead levels. Descriptive data on occupational exposure and the previously described variables are also provided.

| Frequencies of “alcohol consumption” | | | | |
| --- | --- | --- | --- | --- |
| **Alcohol consumption** | **Exposure** | **Observations** | **Relative percentage** | **Cumulate percentage** |
| Never | Yes | 12 | 13.0% | 13.0% |
|  | No | 32 | 34.8% | 47.8% |
| Occasionally | Yes | 15 | 16.3% | 64.1% |
|  | No | 12 | 13.0% | 77.2% |
| 2-3 times a week | Yes | 13 | 14.1% | 91.3% |
|  | No | 2 | 2.2% | 93.5% |
| Daily | Yes | 6 | 6.5% | 100.0% |
|  | No | 0 | 0.0% | 100.0% |

Table S1 - Descriptive data on occupational exposure and alcohol consumption expressed as “frequency of consumption”

| Frequencies of “smoke” | | | | |
| --- | --- | --- | --- | --- |
| **Cigarettes/day** | **Exposure** | **Observations** | **Relative percentage** | **Cumulate percentage** |
| no smoker | Yes | 25 | 27.2% | 27.2% |
|  | No | 24 | 26.1% | 53.3% |
| <10 | Yes | 7 | 7.6% | 60.9% |
|  | No | 4 | 4.3% | 65.2% |
| between 11 and 20 | Yes | 13 | 14.1% | 79.3% |
|  | No | 16 | 17.4% | 96.7% |
| more than 20 | Yes | 1 | 1.1% | 97.8% |
|  | No | 2 | 2.2% | 100.0% |

Table S2 - Descriptive data on occupational exposure and smoke behavior expressed as “cigarettes/day”

| Alcohol consumption and salivary lead levels | Spearman’s coefficient | **0.372** |
| --- | --- | --- |
|  | p-value | **<0.001** |
| Smoke and salivary lead levels | Spearman’s coefficient | 0.053 |
|  | p-value | 0.615 |

Table S3 -Spearman rank correlation between salivary lead levels of the whole sample (N=92), alcohol consumption and smoke
